# Real-time monitoring of an endogenous Fgf8a gradient attests to its role as a morphogen during zebrafish gastrulation

**DOI:** 10.1242/dev.201559

**Published:** 2023-10-03

**Authors:** Rohit Krishnan Harish, Mansi Gupta, Daniela Zöller, Hella Hartmann, Ali Gheisari, Anja Machate, Stefan Hans, Michael Brand

**Affiliations:** ^1^ CRTD – Center for Regenerative Therapies TU Dresden, Technische Universität Dresden, Fetscherstraße 105, 01307 Dresden, Germany; ^2^ PoL – Cluster of Excellence Physics of Life, Technische Universität Dresden, Fetscherstraße 105, 01307 Dresden, Germany; ^3^ CMCB Technology Platform, Technische Universität Dresden, Tatzberg 47-51, 01307 Dresden, Germany

**Keywords:** Fgf8a, Morphogen, Diffusion, Patterning, Neural plate, Zebrafish

## Abstract

Morphogen gradients impart positional information to cells in a homogenous tissue field. Fgf8a, a highly conserved growth factor, has been proposed to act as a morphogen during zebrafish gastrulation. However, technical limitations have so far prevented direct visualization of the endogenous Fgf8a gradient and confirmation of its morphogenic activity. Here, we monitor Fgf8a propagation in the developing neural plate using a CRISPR/Cas9-mediated EGFP knock-in at the endogenous *fgf8a* locus. By combining sensitive imaging with single-molecule fluorescence correlation spectroscopy, we demonstrate that Fgf8a, which is produced at the embryonic margin, propagates by diffusion through the extracellular space and forms a graded distribution towards the animal pole. Overlaying the Fgf8a gradient curve with expression profiles of its downstream targets determines the precise input-output relationship of Fgf8a-mediated patterning. Manipulation of the extracellular Fgf8a levels alters the signaling outcome, thus establishing Fgf8a as a bona fide morphogen during zebrafish gastrulation. Furthermore, by hindering Fgf8a diffusion, we demonstrate that extracellular diffusion of the protein from the source is crucial for it to achieve its morphogenic potential.

## Teaser

**Highlighted Article:** The endogenous distribution of a morphogen directly visualized for the first time in a living vertebrate species and studied to assess the importance of extracellular diffusion in its propagation.

## INTRODUCTION

The induction and organized arrangement of distinct cell types from a field of naïve cells is a fundamental challenge faced by all multicellular organisms during development. One mechanism to achieve this is to use secreted signaling molecules known as morphogens. These are produced from a localized source, distribute through the target tissue in a graded manner and, by virtue of various concentration thresholds, impart distinct positional information to the cells. Cells with the highest exposure to the molecule could then adopt a fate different from the ones with intermediate or lowest exposure, as proposed by Lewis Wolpert in his influential ‘French flag’ hypothesis (Turing, 1952; Wolpert, 1969; Rogers and Schier, 2011; Christian, 2012). Although several embryological and theoretical studies across the 20th century had postulated the existence of such molecules (Morgan, 1901; Lewis, 1904; Spemann and Mangold, 1924; Dalcq, 1938; Stumpf, 1966), the first evidence for morphogens in tissue patterning was provided by the detection of an anterior-to-posterior nuclear gradient of Bicoid in the early *Drosophila* syncytium, which serves to establish anterior-posterior patterns within the developing embryo (Driever and Nüsslein-Volhard, 1988a,b). Subsequently, several extracellular ligands were also identified to enact morphogenic roles in various tissue contexts across Metazoa (Lecuit et al., 1996; Affolter and Basler, 2007; Neumann and Cohen, 1997; Swarup and Verheyen, 2012; Strigini and Cohen, 1997; Briscoe et al., 2001; Dessaud et al., 2008; Chen and Schier, 2001; Schier, 2009).

Fibroblast growth factor 8 (Fgf8, Fgf8a in zebrafish) is a molecule that has been extensively studied for its role as a morphogen. Discovered as an androgen-induced growth factor in a mouse mammary tumor cell line (Tanaka et al., 1992), Fgf8 belongs to a highly conserved family of growth factors, and performs inductive functions during mesoderm formation, neural patterning and organogenesis (Itoh, 2007; Böttcher and Niehrs, 2005; Teven et al., 2014). Fgf8-null mice exhibit improper neuroectoderm patterning and fail to develop through gastrulation, owing to defects in cell migration away from the primitive streak (Sun et al., 1999). Zebrafish *acerebellar* (*ace*) mutants, with no functional Fgf8a protein, lack a midbrain-hindbrain boundary (MHB) and a cerebellum, and are defective in heart and inner ear development (Reifers et al., 1998, 2000; Léger and Brand, 2002). Fgf8, which is expressed at the MHB organizer during early vertebrate development, is thought to act as a long-range signal in patterning the surrounding tissue into the midbrain and rostral hindbrain (Crossley et al., 1996). In the mouse neocortex, Fgf8 is produced from a localized source at the anterior telencephalon, and immunofluorescence staining against Fgf8 has allowed the visualization of its gradient along the anterior-posterior axis. The introduction of ectopic Fgf8 sources by electroporation is further shown to induce target gene expression in a concentration-dependent manner, consistent with its role as a morphogen (Toyoda et al., 2010). In the developing mouse and chick embryo, *fgf8* is expressed in the undifferentiated posterior tip; during axial elongation, it has been suggested that the mRNA is progressively degraded in the differentiated tissue towards the anterior. This generates a posterior-to-anterior mRNA gradient, which could then be translated into a protein gradient eliciting dose-dependent responses (Dubrulle and Pourquié, 2004).

This study focuses on the zebrafish gastrula, where *fgf8a* transcripts are detected at the embryonic margin and its target genes are expressed in increasingly broader domains away from the source (Scholpp and Brand, 2004; Nowak et al., 2011). Our previous work, relying on mRNA micro-injection of fluorescently tagged Fgf8a or transplantation of recombinant Fgf8a-coated beads, has shown that Fgf8a, produced from such artificial sources within the embryo, is capable of forming a protein gradient (Yu et al., 2009; Scholpp and Brand, 2004). Yet, the distribution of Fgf8a, produced from its endogenous locus, has not been studied so far for many reasons. First, morphogens are generally produced in relatively low amounts in an organism and are available in much lower amounts extracellularly, which makes them difficult to detect by immunostaining. Second, fluorescent tagging of molecules at their endogenous loci has historically been difficult in vertebrates due to technical limitations. However, with recent advances in genome engineering, it has now become possible to generate fluorescently tagged fusion constructs of Fgf8a from its endogenous locus (Gaj et al., 2013; Hsu et al., 2014; Barrangou and Doudna, 2016). Together with sensitive imaging platforms and single-molecule analysis, this has enabled us to monitor the endogenous distribution of Fgf8a in real-time in the gastrulating zebrafish embryo and to determine its mode of propagation. We have specifically focused on the developing neural plate of the embryo, where Fgf signaling is known to influence anterior-posterior patterning between early and mid-gastrula stages (Koshida et al., 1998; Kudoh et al., 2002; Grinblat et al., 1998; Woo and Fraser, 1998).

## RESULTS

### Characterization of the Fgf8a-EGFP transgenic line

**Fig. 1.**
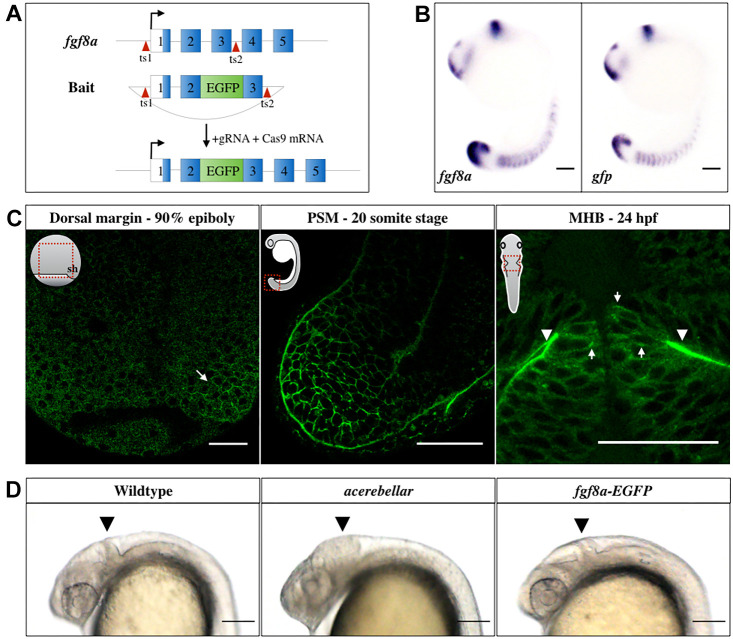
**Generation and characterization of the *Tg(fgf8a:fgf8a-EGFP)* fish line.** (A) Knock-in strategy: EGFP was inserted between exons 2 and 3 of the endogenous Fgf8a locus using CRISPR/Cas9. Exon sequences are shown as numbered blocks, separated by introns. The open reading frame sequence is in blue. Red arrowheads indicate the sgRNA target sites (ts). (B) *In situ* hybridization against *fgf8a* and *gfp* in 16-somite stage (ss) embryos. Scale bars: 100 μm. (C) Fgf8a-EGFP fluorescence in the dorsal embryonic margin (arrow) at late gastrula, PSM at 20 ss, as well as at the basal (arrowheads) and lateral (arrows) sides of the MHB neuroepithelia 24 h post fertilization (hpf). Orientations of embryos are shown schematically in insets. Red rectangles outline the locations imaged. sh, shield. Scale bars: 50 μm. (D) Comparison of homozygous *fgf8a-EGFP* with wild type and homozygous *ace* mutants at 24 hpf. Homozygous viability and normal structure of the MHB (arrowhead) in the *fgf8a-EGFP* transgenics confirms the functionality of the *fgf8a-EGFP* knock-in line. Lateral views are shown. Scale bars: 100 μm.

To visualize the endogenous Fgf8a distribution, we first engineered an *fgf8a-EGFP* knock-in fish line by inserting the EGFP sequence into the endogenous *fgf8a* locus by CRISPR/Cas9 (Irion et al., 2014; Auer et al., 2014; Hoshijima et al., 2016; Kesavan et al., 2018) (see Methods; Fig. 1A). The resulting fusion protein had EGFP integrated immediately after the signal peptide of Fgf8a. This is identical to the construct used in our earlier study, with similar biological functions to the untagged Fgf8a (Yu et al., 2009). *In situ* hybridization against *gfp* faithfully recapitulated the endogenous expression pattern of *fgf8a*, indicating that the fish line could be used as a valid read-out of the gene (Fig. 1B). The Fgf8a-EGFP fluorescence could be visualized in all prominent domains of *fgf8a* expression (Reifers et al., 1998): the dorsal marginal area at late gastrula, in the pre-somitic mesoderm, as well as basolaterally in the midbrain-hindbrain boundary (MHB) neuroepithelium during late somitogenesis stages (Fig. 1C). Homozygous *fgf8a-EGFP* embryos were morphologically indistinguishable from the wild type. They did not lack a MHB or cerebellum and were viable as adults, as opposed to the homozygous *ace* loss-of-function mutants (Reifers et al., 1998), which demonstrated the functionality of Fgf8a in the knock-in line (Fig. 1D).

### Visualization of endogenous Fgf8a-EGFP during gastrulation

**Fig. 2.**
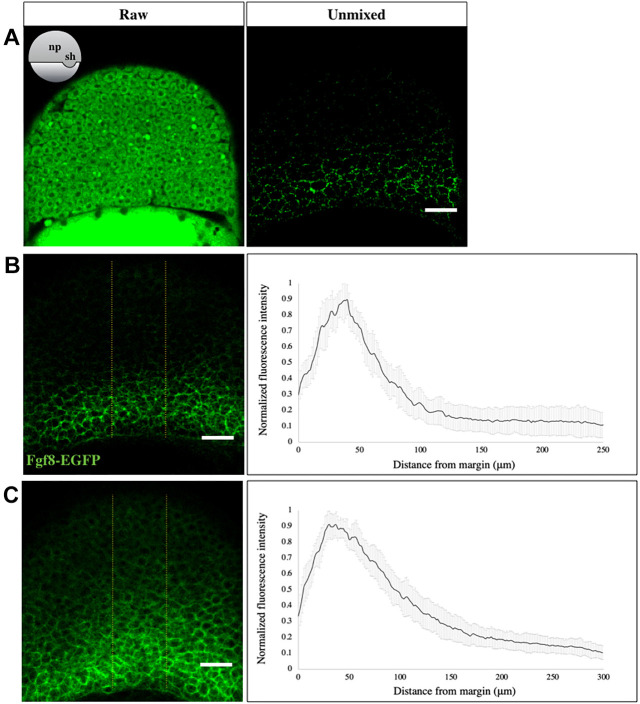
**Fgf8a-EGFP forms a gradient during gastrulation.** (A) EGFP fluorescence in an optical section of the neural plate in early gastrula stage *fgf8a-EGFP* embryos, as visualized using the GaAsP detector, before (left) and after (right) linear unmixing. The orientation of embryos is shown schematically in the inset. np, neural plate; sh, shield. (B,C) Sum-intensity *z*-projected images, derived after linear unmixing (left), used to extract fluorescence intensity profiles in the neural plate (yellow boundaries) at early (B) and mid-gastrula (C) stages. The analysis reveals a graded distribution of Fgf8a towards the animal pole (right). *N*=15 embryos. Scale bars: 50 μm. Data are mean±s.d.

After evaluating the fish line for its fluorescence and biological functionality, we sought to visualize the Fgf8a-EGFP fluorescence profile in the developing neural plate of the embryo from early to mid-gastrula stages. Using conventional confocal microscopy, using a photo-multiplier tube (PMT) for detection, it was not possible to visualize the endogenous Fgf8a-EGFP fluorescence before late gastrulation, owing to its weak expression levels. Therefore, we resorted to a sensitive quantitative imaging protocol using GaAsP hybrid detector for spectral imaging and linear unmixing for subtraction of auto-fluorescent background (Borlinghaus et al., 2012; Zimmermann, 2005) (see Materials and Methods, Fig. S1). This enabled us to visualize the endogenous Fgf8a-EGFP fluorescence in the transgenic embryos starting from early gastrula (∼60% epiboly) (Fig. 2A). By analyzing the signal intensity in the neural plate along the animal-vegetal axis of early and mid-gastrula (∼75% epiboly) staged embryos, we found that Fgf8a-EGFP, secreted from its source at the embryonic margin, forms a graded distribution towards the animal pole (Fig. 2B,C; Fig. S2). Additionally, we also examined the Fgf8a-EGFP fluorescence intensity near the margin, along the dorsal-ventral axis of the embryo at mid-gastrula. This showed that, during gastrulation, Fgf8a-EGFP not only forms a gradient in the animal-vegetal axis but also along the dorsal-ventral axis, with its highest levels at the dorsal shield (Fig. S3). Although the vegetal-to-animal gradient is generated by propagation of the protein from a localized source, the dorsal-to ventral gradient could be a direct manifestation of the dorsal-to-ventral mRNA gradient of *fgf8a*, which has been previously suggested to regulate dorsoventral patterning of the embryo (Fürthauer et al., 1997; Reifers et al., 1998).

### Monitoring Fgf8a-EGFP propagation in the extracellular space

**Fig. 3.**
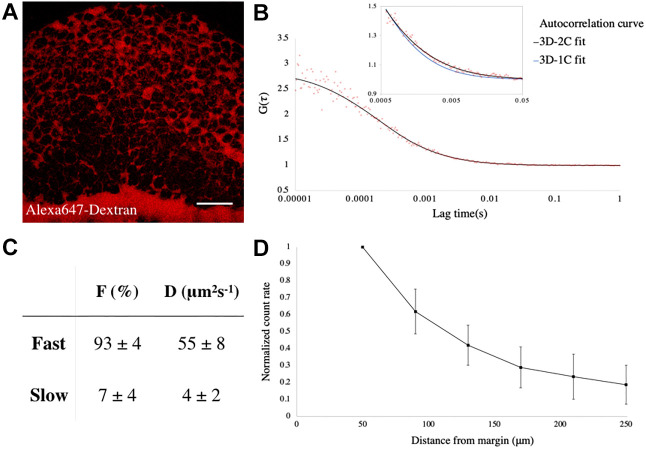
**Fgf8a-EGFP propagates by diffusing via the extracellular space.** (A) Extracellular space (ECS) in an early gastrula embryo, as visualized by Alexa-647-tagged Dextran injection. The orientation of the embryo is as in Fig. 2A. Scale bar: 50 μm. (B) FCS autocorrelation curve from the ECS of a *Tg(fgf8a:fgf8a-EGFP)* embryo fitted with 2-C (black curve) and 1-C (blue curve in inset) 3D-diffusion models. (C) Results from fitting with the 3D-2C diffusion model. F, fraction of each component; D, diffusion coefficient. *n*=52 measurements. (D) Plot of count rate, derived from FCS analysis versus distance from the margin at early gastrula showing graded distribution of Fgf8a in the ECS along the animal-vegetal axis. *N*=20 embryos. Data are mean±s.d.

Having visualized the animal-vegetal gradient of endogenous Fgf8a-EGFP, we then aimed to understand how the molecules, produced at the embryonic margin, traversed towards the animal pole. Although several mechanisms of morphogen transport have been identified, the most prevalent mode of transport is extracellular diffusion (Müller et al., 2013; Stapornwongkul and Vincent, 2021). Our earlier study with exogenous Fgf8a-EGFP showed that it propagates primarily by random diffusion locally via the extracellular space (Yu et al., 2009). However, there could be several artifacts associated with the ectopic expression of a protein, such as aggregation or mislocalization, so it was important to examine the endogenous species (Gibson et al., 2013). To this end, we employed the single-molecule analysis technique of fluorescence correlation spectroscopy (FCS) (Yu et al., 2009; Schwille and Haustein, 2002) in the extracellular space of gastrulating *fgf8a-EGFP* transgenics. The extracellular space was specifically labeled by injecting a non-cell permeable tracer, Alexa647-tagged dextran (Fig. 3A). In general, the FCS auto-correlation curves for extracellular Fgf8a-EGFP fit well with a 3-dimensional (3D) random diffusion model. Many of our measurements (52 out of 97) yielded curves that could not be described by a 3D one-component diffusion model (3D-1C) within the lag times of 0.5-50 ms, but rather by a 3D two-component model (3D-2C) (Fig. 3B; Fig. S4). This suggested the presence of two species of molecules within these confocal measurement volumes: a large proportion (93%) moving with a diffusion coefficient (D_fast_) of 55 μm^2^ s^−1^; and a remaining slow-moving fraction (D_slow_=4 μm^2^ s^−1^) (see Materials and Methods; Fig. 3C). The value of D_fast_ is of the same order of magnitude as that of monomeric EGFP in solution (Petrášek and Schwille, 2008), reflecting free diffusion. The respective proportions of fast- or slow-moving molecules also showed no correlation with distance from the embryonic margin (Fig. S5). Thereafter, to further demonstrate that the endogenous Fgf8a-EGFP forms its gradient by extracellular diffusion, we recorded the FCS fluorescence count rates, which could be used as a direct measure of the molecular concentration of the protein (Yu et al., 2009), from the extracellular space within the neural plate, at varying positions along the animal-vegetal axis. This showed a smooth gradual decrease in protein availability with increasing distances from the embryonic margin (Fig. 3D).

### Deciphering the input-output relationship of the Fgf8a gradient

**Fig. 4.**
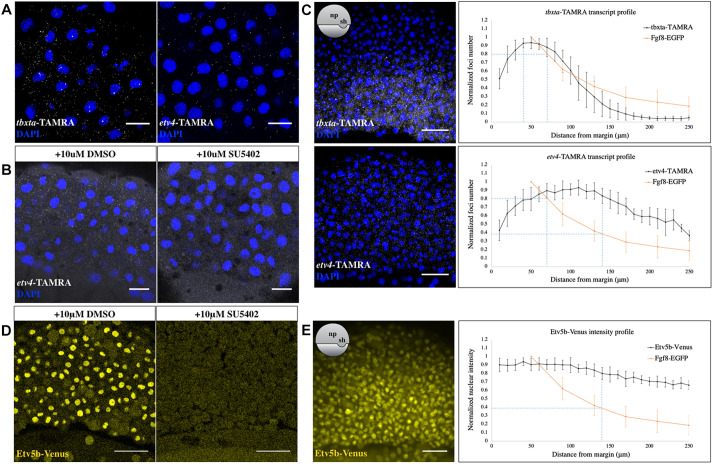
**Input-output relationship of Fgf8a-mediated patterning during gastrulation.** (A) smFISH against *tbxta* and *etv4* in early-gastrula stage embryos, after threshold adjustment for background subtraction. (B) smFISH against *etv4* in DMSO- (left) and SU5402- (right) treated embryos at early gastrula stage. In both A and B, optical sections of lateral views are shown. Scale bars: 20 μm. (C) Maximum-intensity *z*-projected images for smFISH against *tbxta* (top) and *etv4* (bottom) at early gastrula (left), and plot of normalized transcript number versus distance from the margin (right). *N*=13 embryos for *tbxta*, *N*=7 for *etv4*. For A-C, DAPI (blue) was used as a nuclear marker. Overlay with the Fgf8a distribution profile (orange curve) determines its relative extracellular levels corresponding to maximal expression domains of both the transcripts (see blue lines). Peak of Fgf8a-EGFP profile corresponds to an absolute concentration of ∼8 nM. Scale bars: 50 μm. (D) Venus fluorescence in DMSO- (left) and SU5402- (right) treated *Tg(etv5b:etv5b-Venus)* embryos at early gastrulation. Optical sections of laterally mounted embryos are shown. Scale bars: 50 μm. (E) Sum-intensity *z*-projected image of Etv5b-Venus from early gastrula-staged transgenic embryos (left) and analysis of nuclear fluorescence intensity as a function of distance from the margin (right). Overlay with the Fgf8a gradient curve (orange curve) shows its minimum relative abundance in the extracellular spaces that correspond to the peak Etv5b output (see blue lines). *N*=11 embryos. Scale bars: 50 μm. For C and E, exact orientations are shown schematically in insets. Data are mean±s.d.

Next, we sought to decipher how the endogenous Fgf8a-EGFP gradient correlates with discrete domains and levels of target gene expression in the early gastrulating embryo, which would lend additional descriptive support to the morphogenic action of Fgf8a. To achieve this, we first performed single molecule fluorescence *in situ* hybridization (smFISH) (Oka and Sato, 2015) against the Fgf8a target genes *tbxta* and *etv4* (Griffin et al., 1995; Rodaway et al., 1999; Roehl and Nüsslein-Volhard, 2001; Znosko et al., 2010; van Boxtel et al., 2015) in whole-mount early gastrula-staged embryos. Although the *tbxta* foci were specifically detected only within 10 cell tiers from the embryonic margin, there were no locations that were devoid of *etv4* transcripts (Fig. 4A,C). To confirm the specificity of the *etv4* probe set, we inhibited Fgf signaling by treating embryos, from pre-blastula stages, with 10 μM SU5402. No *etv4* foci could now be detected in such embryos at early gastrula, in contrast with the DMSO-treated controls (Fig. 4B). Thereafter, we quantified the expression profiles of both *tbxta* and *etv4*, along the animal-vegetal axis, in the developing neural plate, by counting the number of foci and plotting it as a function of distance from the embryonic margin. This was then overlaid with the Fgf8a gradient data, as obtained using FCS, to determine the various thresholds of the signaling molecule required for the induction of distinct cellular responses. The concentration of Fgf8a-EGFP within the extracellular space at the source domain (C_0_) was calculated from our FCS measurements to be ∼8 nM. The *tbxta* profile was found to closely follow the Fgf8a gradient curve, with the maximum number of foci within 40-70 μm (∼3-5 cell tiers) from the margin, corresponding to a Fgf8a availability of at least 80% of its source concentration (0.8×C_0_). Thereafter, the number of foci decreased steeply, before nullifying at 0.4×C_0_ of Fgf8a, at a distance of ∼10 cell tiers from the margin. For *etv4*, although the transcripts were detected all the way to the animal pole, its peak expression domain was identified to be shifted when compared with that of *tbxta*, with the highest number of *etv4*-positive foci detected within 70 and 140 μm (∼5 and 10 cell tiers) from the embryonic margin. This correlated with extracellular Fgf8a levels of 0.8×C_0_ and 0.4×C_0_, respectively (Fig. 4C). As Fgf signaling is known to be active in the zebrafish blastoderm from 30% epiboly (Reifers et al., 1998), where cells at the animal pole are adjacent to the source, the near-ubiquitous expression of *etv4* that we observe outside the 10th cell tier, up to the animal pole, could result from the lower levels of Fgf8a that we detect using FCS or from previous exposure to the signal and/or could be due to cellular rearrangements occurring over time.

In addition to the smFISH approach, we also used a *Tg(etv5b:etv5b-Venus)* knock-in reporter line to quantitate the expression profile of *etv5b*, another Fgf8a target gene (Roehl and Nüsslein-Volhard, 2001; Znosko et al., 2010). The transgenic line was generated via CRISPR/Cas9-mediated homologous recombination, by which the Venus fluorophore sequence was inserted right before the start codon of the endogenous *etv5b* locus (Fig. S6A). *In situ* hybridization against *etv5b* and *venus* showed identical localization pattern of the transcripts and the fusion protein was found to be localized to the nucleus, consistent with the role of Etv5b as a transcription factor (Fig. S6B,C). This nuclear signal, which was also observed at the YSL, where another Fgf, Fgf3, is expressed (van Boxtel et al., 2018), was completely lost upon incubation with 10 μM SU5402, demonstrating the validity of the transgenic line (Fig. 4D). By measuring the nuclear fluorescence intensity at increasing distances from the embryonic margin, we found a very shallow gradation in protein abundance across the animal-vegetal axis, with peak intensity up to 140 μm (∼10 cell tiers) from the embryonic margin, where 0.4×C_0_ of the morphogen is likely available for signaling (Fig. 4E).

### Manipulation of the endogenous Fgf8a input alters the signaling output

**Fig. 5.**
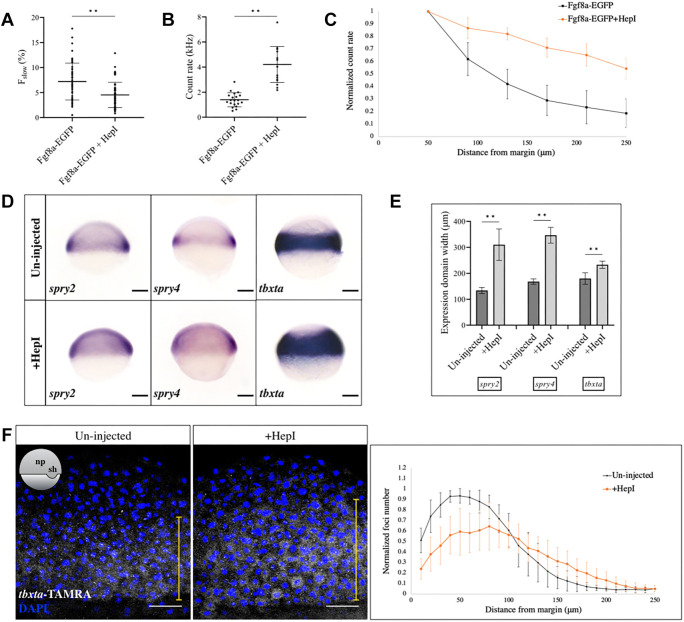
**Manipulation of the Fgf8a input alters the signaling output.** (A) Injection of HepI reduces the proportion of the slow-moving component of Fgf8a-EGFP, as detected by FCS, in the extracellular spaces. *n*=52 measurements for Fgf8a-EGFP, *n*=56 for HepI-injected. **P<0.0001. (B) FCS count rates for Fgf8a-EGFP in the extracellular spaces near the embryonic margin in uninjected and HepI-injected embryos. Count rate is increased upon HepI injection. *n*=20 for uninjected, *n*=17 for HepI. **P<0.0001. (C) Plot of count rate versus distance from margin in control (black) and HepI-injected (orange) cases. *N*=20 embryos for control, *N*=13 for HepI. (D) *In situ* hybridization against *spry2*, *spry4* and *tbxta* in control and HepI-injected early gastrula embryos. Lateral views are shown. Scale bars: 100 μm. (E) Quantification of expression domains of genes as in D, along the animal-vegetal axis. **P<0.0001. (F) smFISH against *tbxta* in uninjected and HepI-injected embryos at early gastrula, and plot of normalized transcript number versus distance from the margin. *tbxta* expression is reduced near the margin and increased in its range away from the margin (yellow line) upon HepI injection. Orientation of embryos is shown schematically in inset. *N*=10. Scale bars: 50 μm. Data are mean±s.d.

We have shown so far that endogenous Fgf8a forms a gradient during gastrulation, and have determined how its extracellular levels correlate with the expression profiles of its target genes. Although these data support the role of Fgf8a as a morphogen, crucial functional evidence for this activity is provided by manipulating the extracellular input and assessing whether this affects the signaling output. Our FCS experiments earlier with the endogenous Fgf8a-EGFP had identified that, although a major fraction of molecules disperses by free diffusion, a minor proportion remains relatively immobile (see Fig. 3C). Based on previous studies, we reasoned that this slow-moving fraction stems from the interaction of these molecules with extracellular matrix constituents, particularly heparan sulfate proteoglycans (HSPGs), which are abundantly localized in the extracellular space as well as at the cell surfaces (Yu et al., 2009; Yan and Lin, 2009; Sarrazin et al., 2011; Gupta and Brand, 2013). Accordingly, the injection of heparinase I (HepI), an enzyme that cleaves the heparan sulfate side chains of HSPGs (Desai et al., 1993), into the *fgf8a-EGFP* transgenics diminished the slow-moving fraction significantly (7.19±3.69% for uninjected; 4.53±2.53% for HepI-injected; mean±s.d.) (Fig. 5A). HepI injections also resulted in an overall increase in the detectable Fgf8a-EGFP levels during FCS in the extracellular space, possibly owing to the dissociation of molecules from cell-surface HSPGs (1.39±0.56 kHz near the embryonic margin for uninjected, 4.21±1.44 kHz for HepI-injected; mean±s.d.) (Fig. 5B). We next examined the effect of HepI injections on the extracellular distribution of Fgf8a-EGFP in the neural plate at early gastrula, by analyzing the FCS fluorescence count rates at increasing distances from the embryonic margin. The application of HepI resulted in a shallower gradient (Fig. 5C; Fig. S7), a finding that was corroborated by GaAsP imaging (Fig. S8). This not only emphasized on the role of Fgf8a-HSPG interactions in shaping its gradient but also yielded us a means with which to manipulate the extracellular levels of endogenous Fgf8a at different positions in the embryo.

To then determine whether this change in Fgf8a-EGFP input results in a concomitant change in the range of signaling outcomes, we first performed *in situ* hybridization against the Fgf8a target genes *spry2* (Fürthauer et al., 2004), *spry4* (Fürthauer et al., 2001) and *tbxta* on early gastrula-staged control and HepI-injected embryos. The expression domains of all three target genes broadened towards the animal pole upon HepI injection (Fig. 5D,E). To further quantitate the effect of HepI-mediated manipulation of the Fgf8a distribution, we used smFISH against *tbxta* in HepI-injected embryos at early gastrula stage and analyzed its foci number as a function of distance from the embryonic margin. The amplitude of its expression profile was found to decrease when compared with the control embryos, possibly owing to the cleavage of HSPG side chains from the cell surface, which affects the ligand-receptor interaction and begets a reduction in signaling strength. Nevertheless, the *tbxta* expression domain itself was found to increase by 2-3 cell tiers, on average, upon HepI injection (Fig. 5F). Thus, flattening the Fgf8a extracellular gradient is found to alter the domains of induction of its downstream targets, providing a functional proof for the morphogenic action of endogenous Fgf8a, consistent with Wolpert's French flag hypothesis (Wolpert, 1969).

### Testing the necessity of diffusion in Fgf8a-mediated tissue patterning

**Fig. 6.**
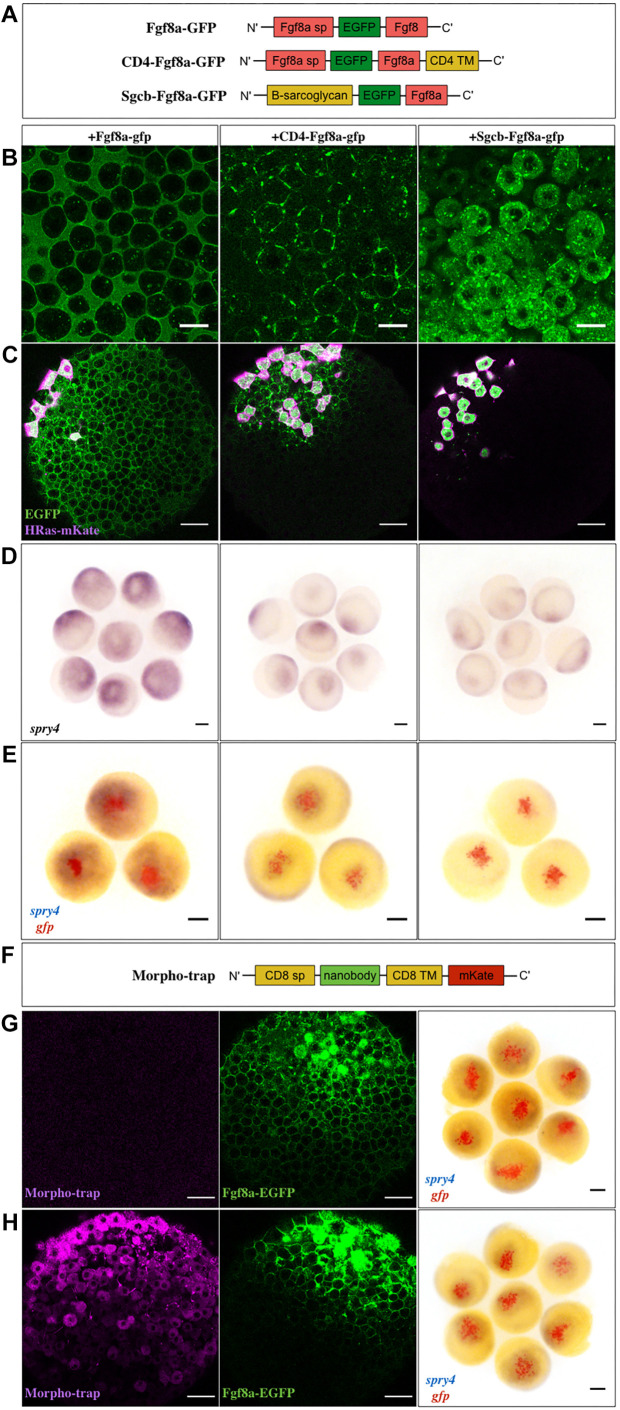
**Extracellular diffusion of Fgf8a is necessary for its morphogenic activity.** (A) The three different versions of Fgf8a were used in this study. sp, signal peptide; TM, transmembrane. (B) Confocal images of late blastula-stage embryos injected with the various constructs at the one-cell stage to assess localization. Scale bars: 20 μm. (C) Generation of ectopic clones of the constructs and imaging at late blastula stages to determine protein spreading. Co-injection with HRas-mKate labels the source cells (magenta). Scale bars: 50 μm. (D) *In situ* hybridization against *spry4* at late blastula after generating ectopic clones as in C. (E) Double *in situ* hybridization against *gfp* (red) and *spry4* (blue) in such embryos at late blastula to depict the ectopic source and target gene induction domains, respectively. Scale bars: 100 μm. (F) Schematic of the Morpho-trap construct. (G,H) Late blastula-stage embryos injected at the 32-cell stage with Fgf8a-EGFP (G) or the Morpho-trap at the one-cell stage followed by Fgf8a-EGFP at the 32-cell stage (H). Confocal images are shown in the first two panels. Scale bars: 50 μm. The third panel depicts double *in situ* hybridization against *gfp* (red) and *spry4* (blue) in such embryos to indicate the Fgf8a-EGFP clone and target gene induction domain, respectively. Scale bars: 100 μm. Animal pole views of embryos are shown throughout.

Finally, we aimed to test the importance of extracellular diffusion in the long-range dispersal and morphogenic activity of Fgf8a. Although extracellular diffusion is at the forefront of the transport models for morphogen gradient formation, the reliability of such diffusion-based gradients in accurately positioning the boundaries of distinct target cell responses and their reproducibility across individuals has been under scrutiny (Kerszberg and Wolpert, 2007). In addition, it has been a matter of debate whether relying primarily on a passive process such as random-walk diffusion can generate suitable gradients within the necessary time frame across a sufficiently large field (Müller and Schier, 2011). Amid this debate, several alternatives to extracellular diffusion have been proposed, among which, directed transport by specialized cellular extensions known as cytonemes has garnered much attention and has been reported for Fgfs in *Drosophila* (Roy et al., 2011; Du et al., 2018). Therefore, we engineered two diffusion-abrogated versions of Fgf8a (Alexandre et al., 2014). EGFP-labeled Fgf8a was attached either to the transmembrane domain of the human T-cell surface protein cluster of differentiation 4 (CD4) (Feinberg et al., 2008) or to the zebrafish full-length transmembrane protein β-Sarcoglycan (Sgcb) (Guyon et al., 2003). Untethered Fgf8a-EGFP, as in our earlier study (Yu et al., 2009), was used for comparison (Fig. 6A). All constructs were micro-injected as mRNA into one-cell stage embryos and imaged at late blastula stages. Although the untethered Fgf8a-EGFP was abundantly secreted into the extracellular space, the CD4-tagged version was primarily anchored to the cell membrane, and the Sgcb-linked protein was located both intracellularly and in patches at the cell membrane (Fig. 6B). Thereafter, we generated ectopic clones of the constructs in developing embryos. A membrane marker, hRas-mKate, was co-injected to precisely label the source cells, and imaging was carried out at late blastula stages to determine the dispersal of the proteins from their respective sources. In the untethered Fgf8a-EGFP-injected embryos, the fluorescent protein diffused away from its source, via the extracellular space, and filled the entire animal pole. In stark contrast, CD4-Fgf8a-EGFP could be found only in cells up to 3-4 cell diameters away from its source, whereas Sgcb-Fgf8a-EGFP was primarily retained within the source cells (Fig. 6C; Fig. S9). Surprisingly, in Sgcb-Fgf8a-EGFP-injected embryos, some protein clusters could also be detected at the tips of mKate-labelled membranous protrusions arising from the source cells (Fig. S10, see Discussion).

Subsequently, we performed *in situ* hybridization against the Fgf8a target gene *spry4* on embryos at late blastula stages. An additional probe against *gfp* was used to precisely detect the source cells. This showed that while the free-diffusing Fgf8a-EGFP induces *spry4* expression at a distance surrounding the source cells, CD4-Fgf8a-EGFP could activate it only in the nearest neighbors, and Sgcb-Fgf8a-EGFP appeared not to have *spry4* activation outside the source cells (Fig. 6D,E). To further corroborate these findings, we used an mKate-labeled anti-GFP nanobody construct, tethered at the membrane by attachment to the mouse CD8 transmembrane region (Morpho-trap) (Rothbauer et al., 2008; Harmansa et al., 2015; Fig. 6F). Expression of this construct in the developing embryos, via mRNA micro-injection at the one-cell stage, was found to abrogate the extracellular diffusion of Fgf8a-EGFP away from an ectopic source and also to reduce its signaling range (Fig. 6G,H; Fig. S11). Thus, we conclude that extracellular diffusion is the predominant mechanism by which Fgf8a propagates over a long range and induces downstream signaling events, thereby ensuring proper patterning of the tissue.

## DISCUSSION

Over 50 years ago, it was proposed that cells, in their undifferentiated states during embryonic development, could be guided towards diverse trajectories by a group of signaling molecules known as morphogens. These molecules, secreted from localized sources within the embryo, were thought to form concentration gradients in their target fields that, by virtue of distinct thresholds, elicit the formation of patterns within a once homogenous environment. Although the existence of many such morphogens has now been established, how these molecules spread to form gradients has been a matter of debate. Here, we aimed to address this by using Fgf8a in the zebrafish developing embryo as a paradigm. Making use of the technical advancements in gene editing and signal detection, we have been able to follow in real-time, the distribution and movement of endogenous Fgf8a molecules during zebrafish gastrulation. We first showed that Fgf8a-EGFP forms a gradient towards the animal pole in the developing neural plate of early and mid-gastrula embryos (Fig. 2B,C). The expression domains of *fgf8a* during early and mid-gastrula stages have earlier been determined to be ∼5 and 8 cell tiers, respectively, from the embryonic margin (van Boxtel et al., 2018; Scholpp and Brand, 2004). This correlates well with the domains of peak fluorescence intensity found in our transgenic samples. The Fgf8a-EGFP fluorescence observed outside these territories therefore results from protein mobility away from its localized source at the margin. Interestingly, we also observed relatively lower levels of Fgf8a-EGFP in the two marginal-most cell tiers. It has been shown previously that Fgf signaling per se is downregulated in these cell tiers via a feed-forward motif involving Dusp4, and that this is crucial for the specification of the endoderm lineage (van Boxtel et al., 2018). However, as it stands, it is unclear whether this motif functions at the level of Fgf8a itself, by downregulating its expression or enhancing its degradation, which would explain our observation.

Thereafter, we identified, using FCS, that the endogenous Fgf8a-EGFP propagates away from its source by diffusing freely through the extracellular space (Fig. 3B-D). The diffusion coefficient (D_fast_=55 μm^2^ s^−1^) was found to be very similar to that reported previously for exogenous Fgf8a-EGFP (53 μm^2^ s^−1^) (Yu et al., 2009). It was important to repeat these measurements with the endogenous molecules as physiological levels of expression cannot be fully guaranteed with ectopic injections, which could have resulted in artifacts such as protein aggregation, mislocalization or non-native protein binding interactions (Gibson et al., 2013; Stapornwongkul and Vincent, 2021). Moreover, the possibility of domain (ectopic versus endogenous) specific differences in transport coefficients could not be ruled out. As in our earlier study (Yu et al., 2009), we also found that a small fraction (7%) of molecules within many of our FCS measurement volumes are relatively immobile/slow moving, which decreased significantly upon HepI injection (Fig. 5A). This implied that the binding of Fgf8a to HSPGs contributes to a significant proportion of this slow-moving component. The remaining fraction could stem from the interaction of ligand with other extracellular matrix constituents, such as chondroitin sulphate proteoglycans or fibronectin, which have been suggested to modulate signaling events in various contexts (Jessen, 2015; Cortes et al., 2009; Muñoz et al., 2006). We also found that the shape of the extracellular Fgf8a-EGFP gradient is heavily influenced by HSPG binding, as it is flattened by the application of HepI (Fig. 5C; Fig. S8). This could be a cumulative effect of two factors: first, under normal circumstances, the binding of molecules to HSPGs in the extracellular space serves to restrict the proportion of molecules in the free diffusive pool at a time; second, HSPGs concentrate the signaling molecules to the cell surfaces and promote ligand binding to their cognate receptors (Yan and Lin, 2009; Sarrazin et al., 2011; Duchesne et al., 2012). Both features could be affected here, as HepI injection not only reduces the slow-moving fraction of Fgf8a-EGFP, but also increases the overall detectable levels of the protein during FCS in the extracellular space (Fig. 5A,B). This now enhances the amount of freely diffusing molecules in the extracellular space that move further, thereby flattening the regular morphogen distribution. Our finding is thus consistent with the ‘hindered-diffusion’ model of morphogen transport, where free diffusion of the morphogen across the embryo is affected by binding interactions (with the extracellular matrix constituents or the cell-surface receptors) and tissue geometry (e.g. presence of cells) (Müller et al., 2013; Stapornwongkul and Vincent, 2021; Ries et al., 2009). Indeed, the relative influences of free diffusion and binding interactions in gradient formation might vary depending on the tissue contexts (Stapornwongkul and Vincent, 2021; Stapornwongkul and Briscoe, 2022). For example, our observation that Fgf8a accumulates basally in the MHB neuroepithelium at a later stage of development suggests that, in an epithelial context, interactions with the extracellular matrix, and the basal lamina in particular, may become more prominent (Fig. 1C). Moreover, in addition to protein diffusion, it is also possible that fluid streaming effects associated with the extracellular matrix and the vegetal movement of the source during epiboly play roles in Fgf8a propagation. However, such large-scale events cannot be studied in the sub-microsecond timescales and sub-femtoliter volume scales employed by FCS.

Does Fgf8a function as a bona fide morphogen during zebrafish gastrulation? Classically, morphogens are considered to disperse away from a localized source in a graded manner and to induce distinct cellular responses according to various concentration thresholds (Wolpert, 1969). Our previous studies using ectopic Fgf8a had shown that it could potentially function as a morphogen by establishing a concentration gradient within its target field, leading to a spatially nested activation of target genes around its restricted source (Yu et al., 2009; Scholpp and Brand, 2004). In this study, we aimed to test this further by working with Fgf8a produced from its endogenous location at endogenous levels. We first determined the extracellular concentration levels of Fgf8a that correlate with the differential expression patterns of some of its target genes at early gastrula (Fig. 4C,E). This served to provide supportive evidence for the role of Fgf8a as a morphogen. Thereafter, we manipulated the extracellular Fgf8a gradient using HepI injections, and found that this altered its target gene induction domains (Fig. 5D-F). Although the *tbxta* territory expanded only by ∼2-3 cell tiers upon HepI injection, *spry2* and *spry4*, the expression of which normally overlaps the *fgf8a* domain, were now induced all the way up to the animal pole. This discrepancy in expansion territories of the targets could stem from the differential competence of cells in activating specific gene expression paradigms, e.g. via distinct gene regulatory networks functional within the cell (Sagner and Briscoe, 2017). Nevertheless, the observation that manipulating the dose curve of Fgf8a affects the subsequent cellular responses attests to its morphogenic role in patterning the zebrafish gastrula.

Although our experiments using FCS dictated that the endogenous Fgf8a propagates by extracellular diffusion, we were mindful of the multiple arguments raised in the field against this mechanism. First, it is predicted that diffusion alone cannot establish a functional gradient quickly enough over large patterning fields (Müller and Schier, 2011). Second, to achieve precise and robust patterning, it is important that the gradients are reproducible across animals. However, gradients formed by diffusion are considered ‘messy’ (Wolpert, 2009). Moreover, it has been estimated for Dpp, a freely diffusing morphogen in the *Drosophila* wing discs, that only a minor fraction of the total morphogen resides in the extracellular space (Zhou et al., 2012). If this is true for Fgf8a, it raises the question of whether diffusion, by itself, of the protein, is capable of generating a functionally relevant concentration gradient within the required time frame over a patterning field as large as the developing embryo. Here, we addressed this query by engineering two diffusion-abrogated versions of Fgf8a and expressing these constructs from ectopic clones within the developing embryo (Fig. 6A-E). We found that the obstruction of extracellular diffusion significantly restricts the signaling domain of Fgf8a. However, it was interesting to observe that the CD4-tethered version progresses up to 3-4 cell diameters away from its source, despite being tethered to the membrane. We also explicitly detected the localization of Sgcb-tethered protein to membranous protrusions emanating from the source cells (Fig. S9). Directed morphogen transport by specialized cellular extensions termed cytonemes is regarded as a prominent alternative to diffusion-based models. The *Drosophila* Fgf family protein Branchless has been identified to be transported in a graded manner, from its source in the wing imaginal disc, to the air sac primordium via cytonemes (Roy et al., 2011; Du et al., 2018). Such cellular extensions have also been discovered in the transport of Wnt8a in zebrafish (Luz et al., 2014; Stanganello et al., 2015). In our case, however, although the Sgcb-version was found to be minimally transported via such extensions, this did not appear to induce observable signaling in the surrounding tissue within the experimental time frame. The CD4-tethered Fgf8a in our study might also be using this mechanism to traverse to the adjacent cell surfaces but we have not been able to capture this. As such tethers have been exogenously engineered, it is not feasible to comment on whether such mechanisms of transport even exist for the endogenous Fgf8a. It has also been shown explicitly in a previous study that ectopic Fgf8-GFP does not localize to such protrusions (Stanganello et al., 2015), which our study is in agreement with. To independently test the notion of extracellular diffusion and support our membrane-tethering experiments, we generated localized sources of untethered Fgf8a-EGFP in embryos expressing a Morpho-trap, which allows the extracellular fraction of Fgf8a-EGFP to be trapped without causing any exogenous modifications to the protein structure. Using this approach, we detected again a diminished signaling range of the molecule (Fig. 6F-H). Indeed, it would have been interesting to now hinder the diffusion of endogenous Fgf8a and evaluate the repercussions on normal patterning events during gastrulation. This would serve to demonstrate the biological relevance of long-distance Fgf8a dispersal during early development in zebrafish, as has been assessed for Wg and Dpp in *Drosophila* wing imaginal discs and a Wnt homolog in *Caenorhabditis elegans* (Alexandre et al., 2014; Harmansa et al., 2015; Pani and Goldstein, 2018). However, such an experiment is not straightforward due to the functional redundancy among Fgfs (Fgf3, Fgf17 and Fgf24) during this time frame and needs to be performed in genetic mutant backgrounds for the other Fgfs (Fürthauer et al., 1997; Koshida et al., 2002; Draper et al., 2003; Fürthauer et al., 2001; Cao et al., 2004). Therefore, we reason that clonal expression of Fgf8a, in this context, presents a more suitable system for studying the effect of diffusion hindrance of Fgf8a alone in tissue patterning. Our results strongly suggest that extracellular diffusion is the predominant mechanism by which Fgf8a can propagate over the necessary distance within the zebrafish blastoderm to generate suitable patterning responses.

**Fig. 7.**
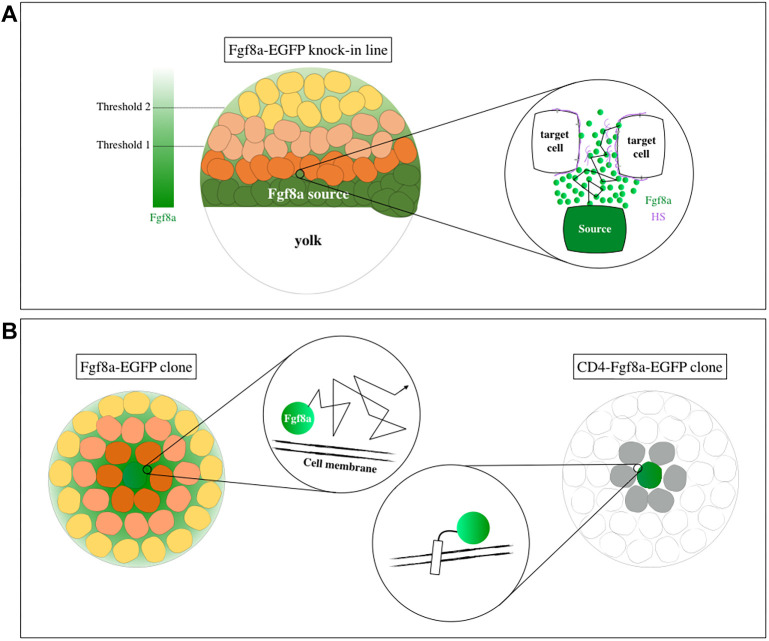
**Schematic summary of the work.** (A) Visualizing endogenous Fgf8a via knock-in reveals its graded distribution during gastrulation (left). Single-molecule FCS detects Fgf8a as an extracellularly diffusing morphogen (enlarged on the right). HS, heparan sulfate. (B) Membrane tethering of Fgf8a in cell clones restricts its signaling range.

Overall, we find here that Fgf8a functions as a bona fide morphogen in the developing neural plate, by establishing a concentration gradient and eliciting threshold-dependent cellular responses, in support of the French flag hypothesis (Wolpert, 1969). We also provide direct evidence that a passive process, such as extracellular diffusion, is sufficient for a morphogen to disperse across its target tissue within the necessary time frame. Although other studies have previously implicated cytonemes as an alternate means of Fgf ligand delivery, we show here that it is crucial for the ligand to diffuse extracellularly to form its long-range gradient (Fig. 7). Future work will aim to hinder the diffusion of endogenous Fgf8a and evaluate its repercussions on normal patterning events during gastrulation. Additionally, it will be key to understand the distribution dynamics of other Fgfs, co-expressed with Fgf8a during gastrulation to fully comprehend the intricate network that is in place to guide the development of a multicellular organism with precision.

## MATERIALS AND METHODS

### Zebrafish husbandry

The fish were raised and housed in the facilities of the Biotechnology Center of TU Dresden (BIOTEC) and Center for Regenerative Therapies Dresden (CRTD), respectively, under conditions described elsewhere (Brand et al., 2002). The adult fish of the required genotype were allowed to mate in specially designed containers filled with fish water. Embryos were collected in Petri dishes filled with E3 medium and stored mostly at 28°C. The staging was carried out according to standard criteria (Kimmel et al., 1995). Live embryos were kept for a maximum of 4 days before discarding or handing over to the facility for raising. AB fish were used as the wild type in this study.

All animal experiments were carried out in accordance with animal welfare laws of the Federal Republic of Germany (Tierschutzgesetz) that were enforced and approved by the competent local authority (Landesdirektion Sachsen; protocol numbers TVV21/2018; DD24-5131/346/11 and DD24-5131/346/12).

### CRISPR/Cas9 transgenesis

For the generation of *fgf8a-EGFP* transgenics, two sgRNA target sites (ts1, GGTGAAGGAATATTTAAAAGTGG; ts2, GGAGAGGAGGGAACGTCTTCAGG; PAM sites are underlined) were chosen as shown in Fig. 1A. sgRNAs and Cas9 mRNA were synthesized as described elsewhere (Jao et al., 2013). The bait sequence, as illustrated in Fig. 1A, was cloned into a pCS2+ vector. 20 pg of this plasmid was injected with 50 pg of both sgRNAs and 150 pg of Cas9 mRNA into one-cell stage wild-type embryos. F0 adults were screened for germline insertion by outcrossing to wild type, extracting the genomic DNA from their progeny and amplifying by PCR, with one primer targeted to EGFP and the other outside the bait (Fwd, GCCTGGCAAGAAATGGGACA; Rev, CAGGGTCAGCTTGCCGTAG). The transgenic line was maintained by outcrossing to wild type. To verify single integration, PCR amplification was carried out from the genomic DNA of animals homozygous for the transgene, with primers (Fwd, GCCTGGCAAGAAATGGGACA; Rev, CTCGACTCCCAAATGTGTCCGT) annealing outside the bait homology arms, which revealed only one band of the expected size. Sequencing of this amplicon revealed no changes other than a 6 bp deletion in the intronic sequence corresponding to ts2.

For the generation of *etv5b-venus* transgenics, a single sgRNA target site (ts, GGAACGCACAGGGATAAACGGGG), with minimal probability of off-target effects according to CRISPRscan.org (Moreno-Mateos et al., 2015), was used (Fig. S6A). The aforementioned protocol was repeated to execute the insertion. F0 adults were screened for germline insertion by PCR amplification from the genomic DNA of progeny, with one primer targeted to Venus and the other outside the bait sequence (Fwd, CAGAGGATTATACAAACGCTGGG; Rev, ACTTGTGGCCGTTTACGTCG).

### Micro-injections

During mRNA micro-injections, 100 pg of the constructs were generally injected either into the cytoplasm of one-cell stage embryos (for expression throughout the embryo) or into a single cell of 32-cell staged embryos (for generation of ectopic clones). For comparison of the various diffusion-hindered Fgf8a constructs (as in Fig. 6B,C), 100 pg of Fgf8a-EGFP mRNA was injected as control, and equimolar amounts of the other versions were injected. For the Morpho-trap experiment in Fig. 6H, 250 pg of the construct was injected at the one-cell stage, followed by injection of 100 pg Fgf8a-EGFP into a single cell at 32-cell stage. For the visualization of extracellular space as in Fig. 3A, 0.2 nl of 5 μM solution of Alexa Fluor 647-labeled Dextran (Invitrogen) was injected into the animal pole of sphere-staged embryos. In the case of heparinase I injections (as in Fig. 5), 0.2 nl of 1 unit/μl solution of heparinase I (Sigma-Aldrich) was injected five or six times at various positions in sphere-stage embryos to ensure an even distribution of the enzyme.

### SU5402 treatments

Embryos were manually dechorionated and treated from pre-blastula stages with 10 μM SU5402 or DMSO, and fixed or imaged at early gastrula.

### *In situ* hybridization

Whole-mount *in situ* hybridization was carried out as described previously (Reifers et al., 1998). Probes were prepared from linearized plasmid templates using the DIG/Flu RNA labeling mix (Roche). 1:4000 of anti-dig-AP and 1:1000 of anti-flu-AP antibodies (Roche) were used for the staining procedure. BM purple (Roche) and FastRed substrate (Sigma-Aldrich) were used to develop the blue and red staining, respectively.

A previously established protocol (Oka and Sato, 2015) was used to perform smFISH. While oligos against *etv4* were designed using the Stellaris Probe Designer, an available probe set (Oka and Sato, 2015) was used for *tbxta*. TAMRA-labeled (at 3′ end) probe sets for both genes were synthesized from Biosearch technologies. A final probe concentration of 250 nM was used for detection (see Table S1).

### Imaging

Live or fixed embryos were appropriately mounted and oriented either in 1% low-melting agarose in E3 or 70% glycerol (whole-mount *in situ* samples). All stereo-microscope derived images were taken using an Olympus MVX10 MacroView fluorescence microscope. Confocal images were acquired using a 40× C-Apochromat NA1.2 water immersion objective of the Zeiss LSM780/Confocor3 microscope, at a room temperature of 22.5°C.

### GaAsP imaging protocol

For visualization of endogenous Fgf8a-EGFP, confocal imaging was implemented using the GaAsP hybrid detector (Borlinghaus et al., 2012). This allowed for simultaneous acquisition of spectral information from the samples, which was coupled to the mathematical technique of linear unmixing using ZEN (Zeiss) to separate the various spectral profiles emanating from the sample (Zimmermann, 2005). *Tg(bactin:hRas-EGFP)* embryos, in which EGFP is found in abundance, and gastrula-staged wild-type embryos (from wild-type siblings of the transgenic fishes), were imaged using the GaAsP to obtain the specific emission spectrum of EGFP and the various spectra corresponding to background autofluorescence, respectively. See Fig. S1 for an example of such spectral profiles. These were subsequently used as references for linear unmixing of images derived from *fgf8a-EGFP* to subtract the pixels corresponding to background autofluorescence and visualize only those specific to EGFP.

### Fluorescence correlation spectroscopy

Fluorescence correlation spectroscopy (FCS) measurements were performed using an in-built FCS setup of a Zeiss LSM780/Confocor3 microscope, with the 40× C-Apochromat NA 1.2 water immersion objective, at a room temperature of 22.5°C. A 488 nm Argon laser (laser power-15 μW) was used for excitation and a BP 505-540 IR emission filter was used for detection, with the pinhole set to 1 AU. On each day of measurement, the microscope was calibrated for the correction collar and pinhole alignment using 50 μl of 15 nM Alexa fluor 488 (A488). For the experimental samples, each position of measurement was manually selected by acquiring a confocal image of Alexa647-Dextran using the appropriate setting to visualize the ECS and five repetitions of 10 s-long FCS measurements were taken.

Subsequent analysis was carried out as described previously (Yu et al., 2009). In short, the curves obtained for A488 were fit with a three-dimensional one-component diffusion model (3D-1C) using ZEN (Zeiss) and the average dwell time (τ_D_) for the dye in the observation volume was determined. The triplet fraction and triplet relaxation time were fixed at 10% and 30 μs, respectively, while the structural parameter (S=ω_o_/z_o_, where ω_o_ and z_o_ are the 1/e^2^ radii of the detection volume in the lateral and axial direction, respectively) was allowed to fit freely. The average value obtained for τ_D_ was then used to calculate ω_o_, using the equation D=ω_o_^2^/4τ_D_, where D is the diffusion coefficient (literature value) of A488 in solution. For all experimental samples, the FCS auto-correlation curves were fit with either 3D-1C or 3D-2C diffusion models and the efficiency of fitting was compared. Again, the triplet fraction and triplet relaxation time were fixed at 10% and 30 μs, respectively, while the structural parameter was fixed as the average obtained for A488 (∼5). The dwell times obtained from the appropriate fit and the lateral radius of detection volume, determined earlier, were used to calculate the diffusion coefficients of the molecules. FCS measurements within the extracellular space in the source domain were used to calculate Fgf8a-EGFP concentration (C_0_) according to the formula C_o_=N_p_/V_eff_, where N_p_, the number of particles, is given by the fit, and the confocal measurement volume V_eff_ = π^3/2^ω_o_^2^ z_o_.

### Fluorescence intensity analysis

Confocal images were analyzed for overall fluorescence intensity using Fiji/ImageJ. Optical sections for a given sample were sum-intensity projected along the *z*-axis and the intensity profile across a 100 μm wide region was extracted. This was then plotted as a function of the distance from the embryonic margin while monitoring endogenous expression, or from the source boundary wherever clonal analysis was performed. For each embryo, the profile was thereafter normalized to the maximum intensity value for that sample.

For analysis of nuclear fluorescence intensity, confocal *z*-stacks were processed using a denoising filter, followed by nuclear segmentation using a blob-finder analysis operator (diameter, 5 μm; threshold, 20%), both performed in Arivis. All blobs within a region 100 μm in width, corresponding to the neural plate, were selected and their mean signal intensities were extracted. Such values were binned in 10 μm intervals, along the animal-vegetal axis, and plotted as a function of distance from the embryonic margin. The maximum of the binned values for each sample was used for normalization within that embryo.

### Gradient analysis using FCS

FCS measurements were carried out in the extracellular space at increasing distances from the embryonic margin, and the fluorescence count rates (CRs) were recorded. The average CR corresponding to background autofluorescence was subtracted by taking FCS measurements in wild-type embryos. The CR values within a given sample were normalized to the maximum CR obtained for that sample. Resulting values from several such samples were binned in 40 μm intervals, averaged and plotted as a function of distance from the margin.

### smFISH foci analysis

Individual smFISH foci were segmented using the Blob finder analysis operator in Arivis. The blob radius was set to 1 μm, which was found to efficiently detect the most foci. A threshold <5 was always applied but re-adjusted for each image manually to facilitate the detection of the maximum number of spots that could be identified visually. A rectangular region of interest, corresponding to the neural plate, was selected across the animal-vegetal axis and divided into 10 μm blocks using a custom-written Python script. Blob counts within each 10 μm bins were recorded and plotted as a function of distance from the embryonic margin. For analysis of endogenous expression profiles, normalization was carried out within each sample to the maximum of the binned value for that sample. For analysis of *tbxta* expression in heparinase I-injected samples, the average of the maximum from all control samples was used for normalization.

### Statistical tests

Sample sizes were chosen according to standards in the field. Throughout the paper, *N* represents number of embryos, *n* (in case of FCS measurements) represents number of measurements, always at distinct locations. In order to determine whether two datasets were significantly different from each other, a *t*-test was performed using GraphPad. It was assumed that the datasets follow a normal distribution and an unpaired two-tailed *t*-test (with equal variance) was applied to compute the *P*-values. *P*<0.05 was considered to be statistically significant. To check for correlation in Fig. S5, a Pearson correlation coefficient (r) was computed using GraphPad.

## Supplementary Material

10.1242/develop.201559_sup1 Supplementary information
