# Supplementary material for: Real-time monitoring of an endogenous Fgf8a gradient attests to its role as a morphogen during zebrafish gastrulation
Source: Development. 2023 Oct 3;150(19):dev201559. doi: 10.1242/dev.201559 (PMC10565248; doi:10.1242/dev.201559)
Supplement: Supplementary information [file develop-150-201559-s1.pdf]

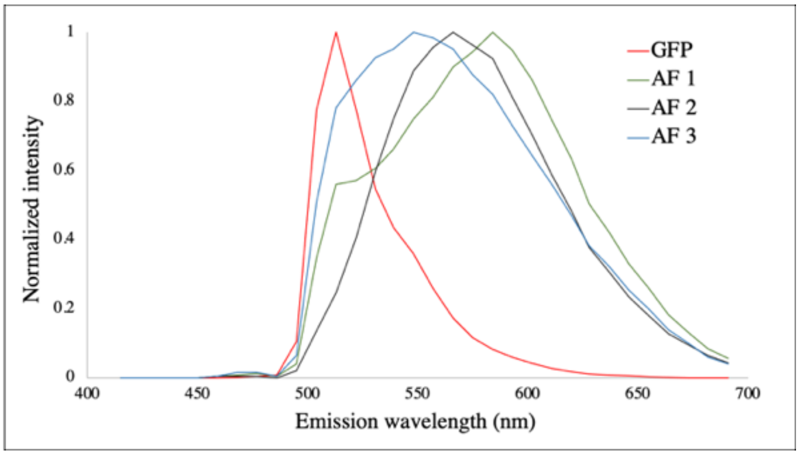

Fig. S1. Spectra for linear un-mixing. Emission spectra profiles corresponding to EGFP and the various autofluorescence components (AF 1-3), extracted by imaging EGFP positive and wildtype embryos respectively, using the GaAsP detector, and subsequently used for linear unmixing in Fig. 2.

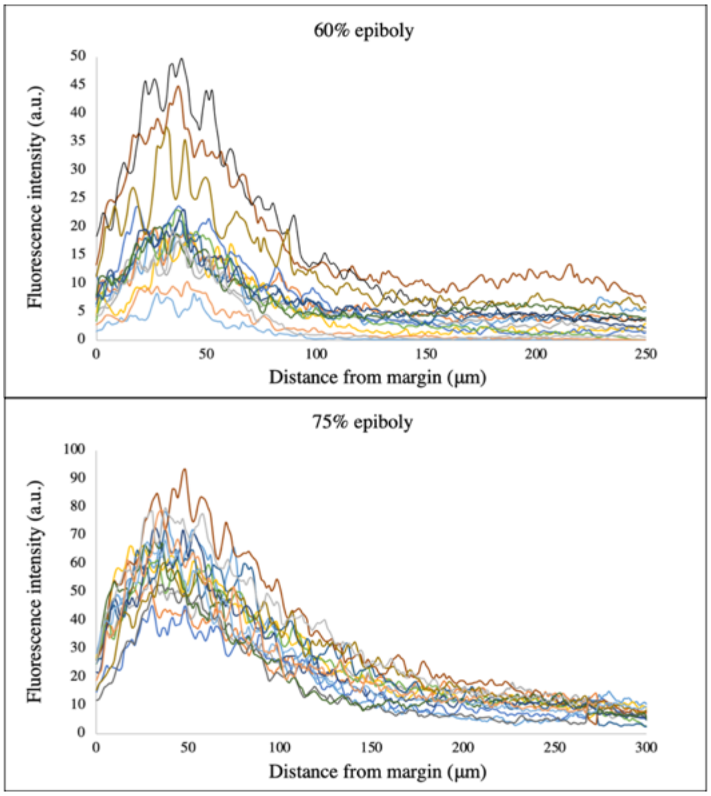

Fig. S2. Fgf8a-EGFP gradients along the animal-vegetal axis during gastrulation. Fluorescence intensity (absolute) profiles for Fgf8a-EGFP along the animal-vegetal axis at the early (top) and mid-gastrula (bottom) stages. Each curve corresponds to a single embryo.

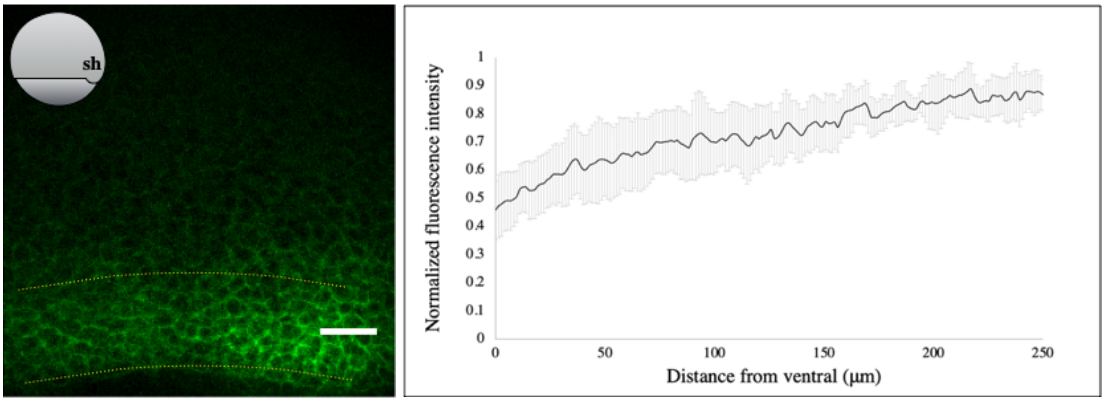

Fig. S3. Dorsal-to-ventral gradient of Fgf8a-EGFP during gastrulation. Sum-intensity z-projected image of Fgf8a-EGFP in mid-gastrula staged embryos (left) and normalized fluorescence intensity analysis at the embryonic margin (between the yellow boundaries) from the ventral to dorsal side of the embryo (right). N=10. Orientation of the embryo is shown in inset. sh-shield. Scale bar-50 µm. Data are mean±s.d.

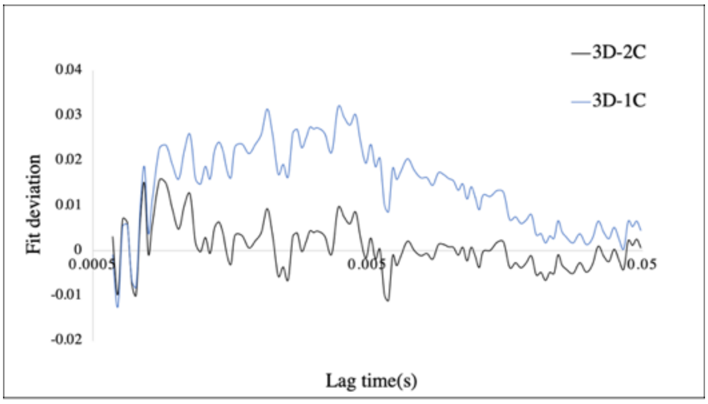

Fig. S4. Deviation of fitted models from the FCS autocorrelation data. Deviation of 3D- 2C (black) and 3D-1C (blue) model fits from the FCS autocorrelation data within the lag times of 0.5-50 ms. The average deviation from 10 measurements is shown in the graph.

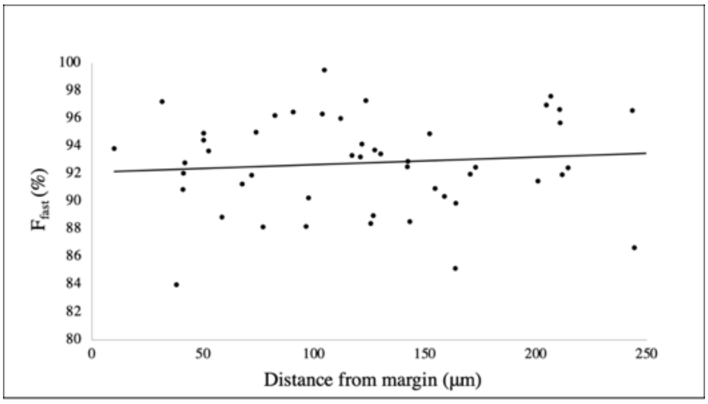

Fig. S5. Plot of F_{fast} vs distance from the margin. The proportion of fast moving Fgf8a-EGFP molecules determined using FCS at early gastrula, vs distance of measurement from the embryonic margin. Dots represent individual datapoints. Note the lack of correlation between the two parameters (Pearson correlation coefficient, r = 0.0619).

Development • Supplementary information

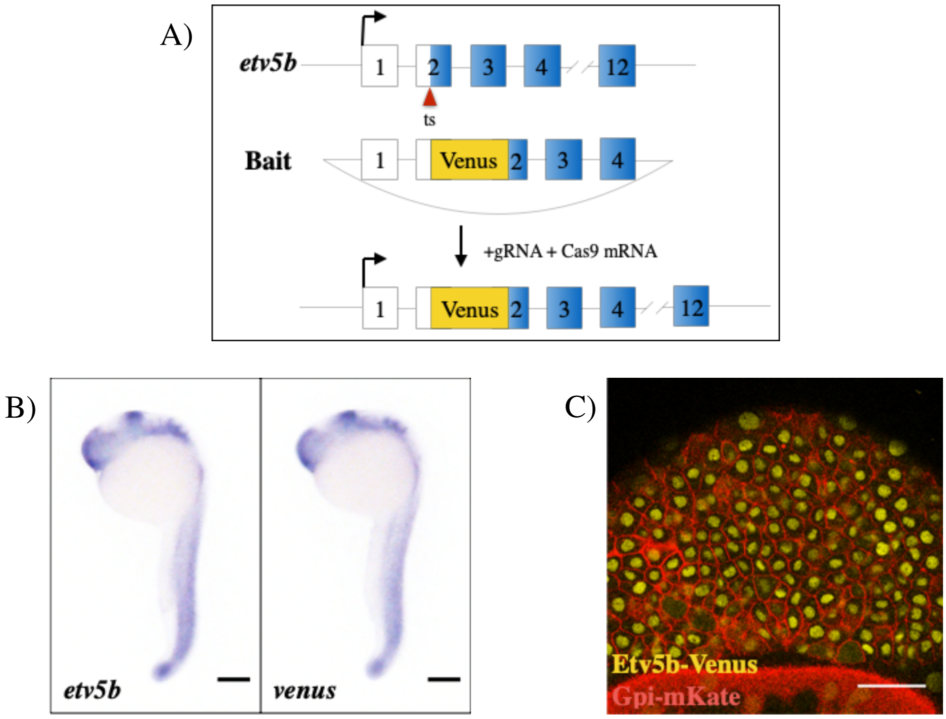

Fig. S6. Generation of *Tg(etv5b:etv5b-Venus)* fish line. A) Knock-in strategy for generation of *Tg(etv5b:etv5b-Venus)*. Venus was inserted right before the start codon of *etv5b* endogenous locus using CRISPR/Cas9 mediated homologous recombination. Exon sequences are shown as numbered blocks, separated by introns. The open reading frame sequence is coloured blue. Red arrowhead denotes the sgRNA target site (ts). **B)** *In situ* hybridisation against *etv5b* and *venus* in 24hpf embryos. Scale bars-100 µm. **C)** Nuclear localisation of Etv5b-Venus fluorescence (yellow) visualised at early gastrula. Optical section of a laterally mounted embryo is shown. Gpi-mKate is used as membrane marker (red). Scale bar-50 µm.

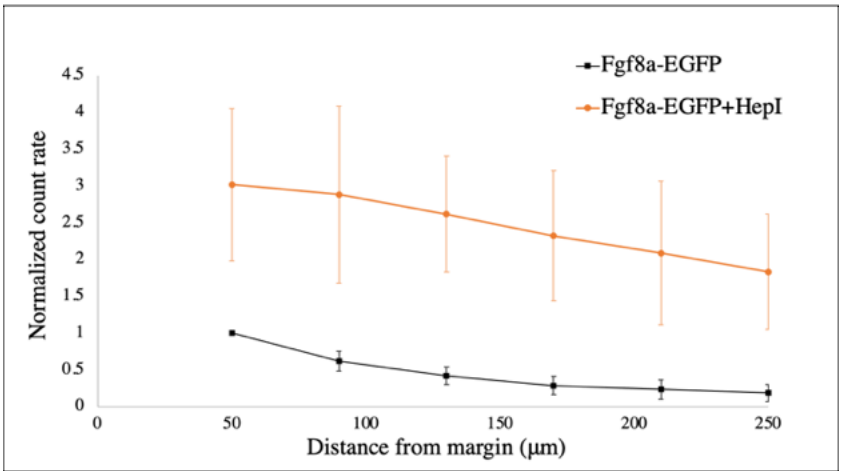

Fig. S7. HepI injection alters Fgf8a-EGFP levels in the extracellular space. Plot of count rate, as measured by FCS, vs distance from margin in control (black) and HepI (orange)-injected embryos. Values for HepI are normalised to average of maxima for control embryos. N=20 for control, N=13 for HepI. Data are mean±s.d.

Development • Supplementary information

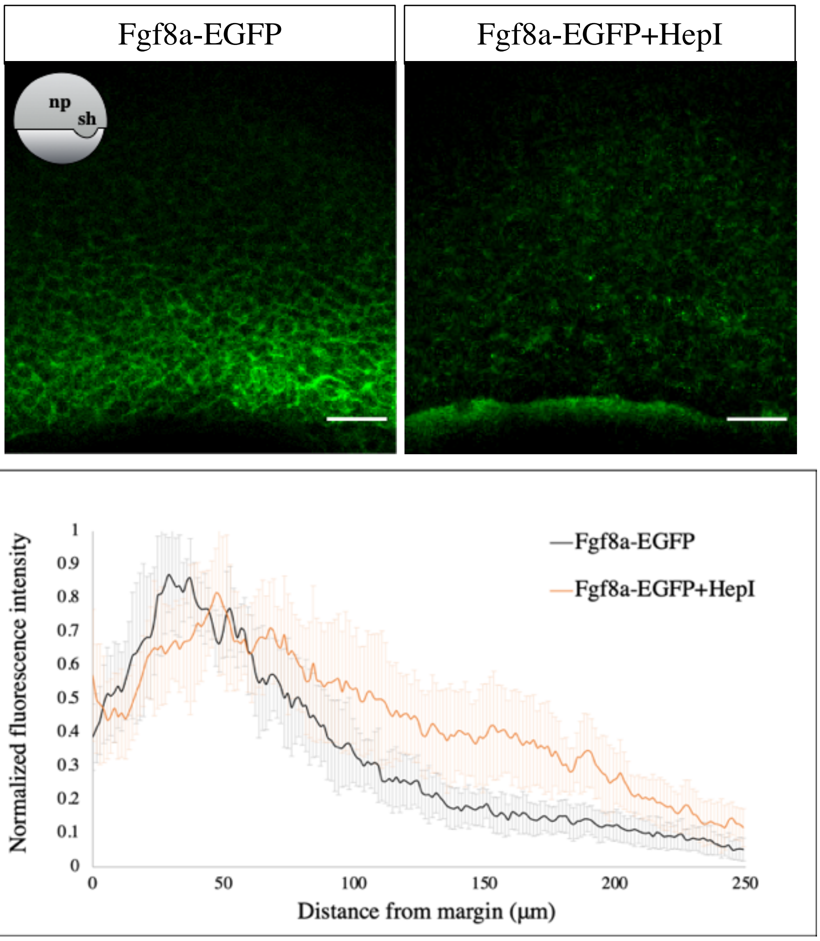

Fig. S8. HepI injection results in a shallower Fgf8a gradient. GaAsP-generated images (after linear unmixing) of un-injected and HepI-injected *fgf8a-EGFP* embryos (top panel) reveals shallower EGFP signal upon HepI injection. Analysis of fluorescence intensity (normalised for each embryo) is shown in the bottom panel. N=6 for control, N=8 for +HepI. Orientation of the embryos is shown in inset. Scale bars-50 µm. Data are mean±s.d.

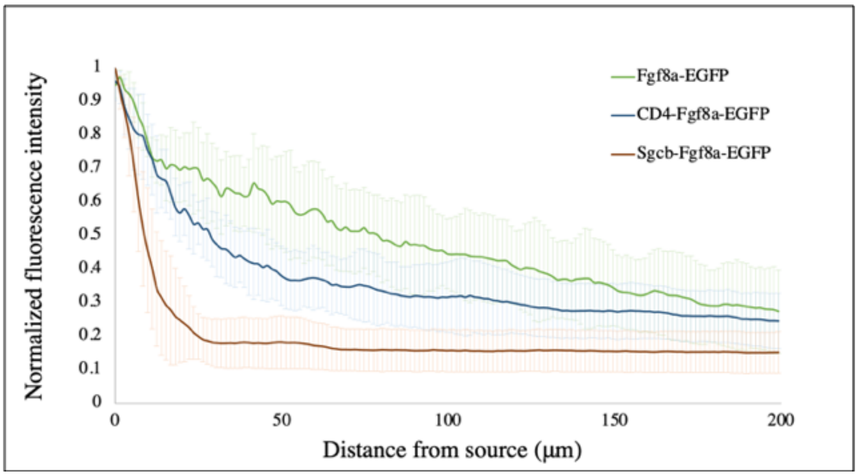

Fig. S9. Hindering Fgf8a diffusion using membrane tethers. Fluorescence intensity profiles for the various Fgf8a constructs away from their ectopic clones at the late-blastula stage. N=10 for each. Data are mean±s.d.

Development • Supplementary information

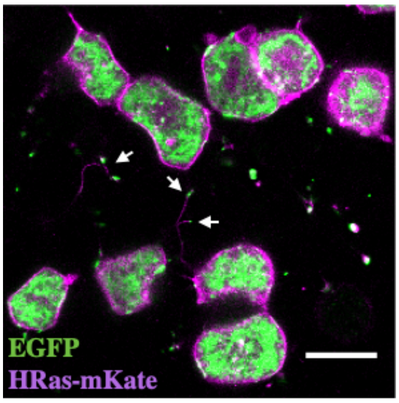

Fig. S10. Transport of Sgcb-Fgf8a-EGFP via membrane protrusions. Co-localisation (arrows) of Sgcb-tethered Fgf8a with mKate positive membranous protrusions generating from the ectopic source cells. Scale bar-20 µm.

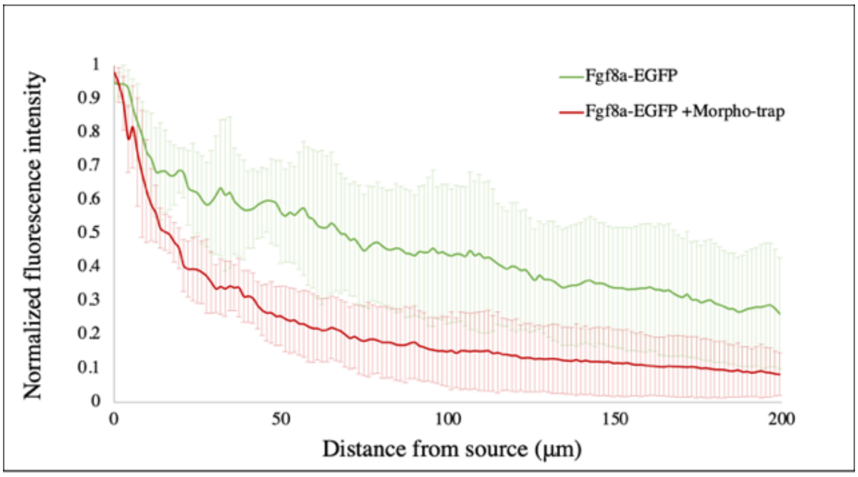

Fig. S11. Hindering Fgf8a diffusion using Morpho-trap. Fluorescence intensity profiles for Fgf8a-EGFP away from its ectopic source in Morpho-trap injected vs un-injected embryos. N=5 for each. Data are mean±s.d.

Development • Supplementary information

Table S1. Probe sets for smFISH

RNA	Probe set (5′-3′)
etv4	CCATCCATCTTATAATCCAT, AGTATAAGGCACTTGCTGGT, ATTTCCTTGCGACCTATTAG, TCAACAGTCTATTTAGGGGC, ATGTATTTCCTTTTTGTCGC, CTGAGGGGGTAATTCTGCGT, CCTGAAAGAGGTCTTCAGAT, GTCTCCTGAAGTTGGCTTAA, GGAACTTGAGCTTCGGTGAG, AACAAACTGCTCATCGCTGT, CACTGAGTTCTCTGAGTGAA, TTCTTAATCTTCACAGGCGG, TAGCTGAAGCTTTGCTTGTG, TAAAGGCACTGCTCTCCATT, TCTTCTGCTCATAGGCACTG, CTGGACATGAGCTCTTAGAT, TTGGGGGAATAATGCTGCAT, TGAGGGTGGATTCATATACC, TCATGGGGTAACTGTGGCTG, CGGAAGGGAACCTGGAACTG, AAGCGAATGGTGGGCACATC, AGAGTGTTGCCGATGGTAAC, GAGGATAAGGCAAGCAGGGG, TCCTGCTTAAAGGTCTGCTG, GTCATACAGAGGGTCCATGT, AGGAAACCTCTGAGGCTGTG, TCCTGTTTGACCATCATATG, CAGGTTCGTAAGTGTAGTCG, TGTGATGGTACATGGATGGG, TGTGCTGTGGGTTGGAGTAG, TCAAACATGTAGCCTTCACT, TGGCACAACACGGGAATCAT, TCACCTCACCTTCAAATTTC, ACCTTCACGAAACACACTGC, TAGTTGAAGTGAGCCACGAC, GAAGGGCAACCAAGAACTGC, ATGCGATGAAGTGGGCATTG, TCAATGAGTTTCATGCCACG, TTGTCATAGTTCATGGCTGG, GTAACGCAAAGAGCGACTCA, TTTTGCATAATTCCCTTCTC, AAACATAACGCTCACCAGCC, CTTCTGGCTCACACACAAAT, TTGTCGGGAAAAGCCAAGGA, GTTGACGTAGCGCTCAAATT, CTCATCGAGGTGGGACAGTG, GGTTCGGGAGGGTAAGAAAC, TACATGTAGCCTTTGGAGTA
tbxta	TCGGGACTTGAGGCAGACAT, CTAAGGAGATGATCCAGGCG, TTCTGAAATTCGCTCTCCAC, AAGTTTAATATCCCGCTCGG, CACAACTCCGCGTCTTCAAG, GGTGAGCTCTTTAAATTTGG, CCAGTCTTGGTGACAATCAT, TGAGCACGGGAAACATTCGT, CATTGCATTAGGGTCGAGAC, CAAAATCCAGCAGGACCGAG, ATTCACCGTTCACGTATTTC, GTGGATGTAGACGCAGCTCG, AAGATACGGGTGCTTTCATC, TTGGAGAGTTTGACTTTGCT, TAACATAATCTGTCCTCCTC, GGGTTCGTATTTGTGCAATG, CCGACTTTCACGATGTGTAT, CTGACTGCTGATCATTTTCT, GCAATAAACTGTGTCTCAGG, TCTCTTCATTCTGATATGCT, TGTTTGATTTTCAGAGCGGT, CATCGAGGAAAGCTTTGGCA, TTGTGGTCACTTCTCTCTTT, CAGATTGCTGGTTGTCAGTG, CAGCCACCGAGTTGTGAATA, CATTGAACTGAGGAGGGCTG, TACGAACCCGAGGAGTGAAC, CAAGCTGGAGTATCTCTCAC, TAATGGCTGGGATATGGAGC, TAGTTATTGGTGGTAGTGCT, AGACTTCCGGAAGAGTTGTC, TGACCAGCTGTCATGAGACG, TGTTGGAGGTAGTGTTTGTG, TGCAACTGACCACAGACTTG, TAATGGAGCCCGATGCTGAG, GCGTAGGAACTGAGATGTCA, CCGAGTAGGACATCGAAGAA, GAGGGAGAGGACACAGGCAG, TAGGCCTGGATCGTACATTG, CGATGGAGCTCTCGAACTGG

Development • Supplementary information
